# Structural Requirements for the Binding of a Peptide to Prohibitins on the Cell Surface of Monocytes/Macrophages

**DOI:** 10.3390/ijms23084282

**Published:** 2022-04-13

**Authors:** Qindong Zhang, Anniken Olberg, Mouldy Sioud

**Affiliations:** 1 Division of Cancer Medicine, Department of Cancer Immunology, Oslo University Hospital, University of Oslo, Ullernchausseen 70, 0379 Oslo, Norway; qindong.zhang@rr-research.no (Q.Z.); anniken.olberg@rr-research.no (A.O.); 2 Department of Pharmacy, Faculty of Mathematics and Natural Sciences, University of Oslo, P.O. Box. 1068, Blindern, 0316 Oslo, Norway

**Keywords:** peptides, macrophages, targeted delivery, prohibitins, tumor microenvironment, phage display

## Abstract

The screening of phage peptide libraries resulted in the identification of a sequence (named NW peptide, NWYLPWLGTNDW) that specifically binds to human monocytes and macrophages. Although the NW peptide can be used for the targeted delivery of therapeutics without knowledge of its receptor(s), the identification of-its binding partners will support future clinical applications-Here, we used the biotinylated NW peptide for cross-linking cell surface receptor(s) on live cells or as bait in pull-down assays with membrane proteins isolated from monocytes or human THP-1 cells differentiated into macrophages. Proteomic analysis of the captured proteins identified cell surface prohibitins (PHB1 and PHB2) and modified albumin as binding partners. Using flow cytometry and pull-down methods, we demonstrated that PHB1 and PHB2 interact directly with the NW peptide. Confocal imaging showed co-localization of the peptide with PHB1 on the surface of monocytes. Single replacement of either tryptophan or leucine with alanine completely inhibited binding, whereas the replacement of asparagine at position 1 or 10 and aspartic acid at position 11 with alanine did not affect the binding of the peptide variants. Neutral amino acid replacement of tryptophan at positions 2, 6, and 12 with tyrosine or phenylalanine also abolished the binding, implying that the indole ring of tryptophan is indispensable for the NW peptide to bind. Overall, the data suggest that membrane-associated prohibitins might be a useful target for the delivery of therapeutics to monocytes/macrophages and that tryptophan and leucine are key residues for peptide binding.

## 1. Introduction

Therapeutic advances in cancer immunotherapy have quickly emerged over the past few years [1,2]. However, only a small fraction of patients respond to the currently available therapeutic options, in part due to the immunosuppressive tumor microenvironment (TME) and antigen loss [3,4]. Present clinical treatment strategies generally do not incorporate the targeting of immunosuppressive cells and/or their progenitors. Although the exact composition of the TME varies among cancer types, tumor-associated macrophages (TAMs) have been identified as a critical cell population across the majority of cancers, playing a major role in the progression of solid tumors [5]. In general, macrophages are classified into two main groups: classically activated M1 macrophages (pro-inflammatory) and alternatively activated M2 macrophages (anti-inflammatory) [6,7]. Polarization is a process where macrophages exhibit vastly different gene expression profiles and functions in response to environmental signals. The polarity of a macrophage population toward M1 or M2 is not fixed but can change depending on the stimuli received from the TME [8]. TAMs mostly originate from blood monocytes, with a minority deriving from tissue-resident macrophages [6]. In most tumors, infiltrating monocytes are educated by the TME to adopt supportive roles in promoting tumor progression. In solid tumors, TAMs that acquire pro-tumoral M2-phenotype can account for up to 50% of the tumor mass [9]. The abundance of TAMs is associated with poor prognosis in the vast majority of solid tumors, including lung, breast, prostate, ovarian, glioma, and cervical cancers [10,11]. In addition to damping anti-tumor immunity, M2 macrophages also induce resistance to current therapies, including chemotherapy, radiotherapy, and checkpoint inhibitor-based immunotherapy [10,12,13,14,15]. Thus, a strategy that improves the targeted killing of TAMs and/or their monocyte progenitors is needed. To interfere with the pro-tumoral function of TAMs, two main strategies can be applied: (i) to cut off their replenishment by targeting circulating monocytes and (ii) to selectively deplete TAMs in the tumor microenvironment.

Cell-targeting peptides, including penetrating peptides, are a group of small peptides that can bind and translocate into specific cell types [16,17,18,19]. As such, they are particularly appropriate for the targeted delivery of therapeutics. By the use of random peptide phage libraries, we have isolated a number of short peptides that bind specifically to human monocytes and macrophages, as well as monocyte-derived dendritic cells, when compared to lymphocytes (e.g., T cells, B cells, and NK cells) [20,21]. One of the selected peptides, named NW peptide, was fused to various lytic peptides and shown to kill monocytes/macrophages [22]. This suggests that the receptor(s) of this peptide could be useful target(s) for the delivery of therapeutics to these cells. Although peptides selected from phage display have been used for drug delivery and imaging without knowledge of their cellular receptors, moving any peptide toward clinical use will be facilitated by the understanding of its interaction with target cells, as well as the identification of the cell surface receptor(s). In the present study, we report on the identification of amino acids that are critical for the NW peptide to bind monocytes/macrophages. Moreover, we uncovered membrane-associated prohibitins (PHB) 1 and 2 as the binding receptors.

## 2. Results

### 2.1. Cross-Linking Experiments Identified Modified Albumin as an Interacting Protein

**Figure 1:**
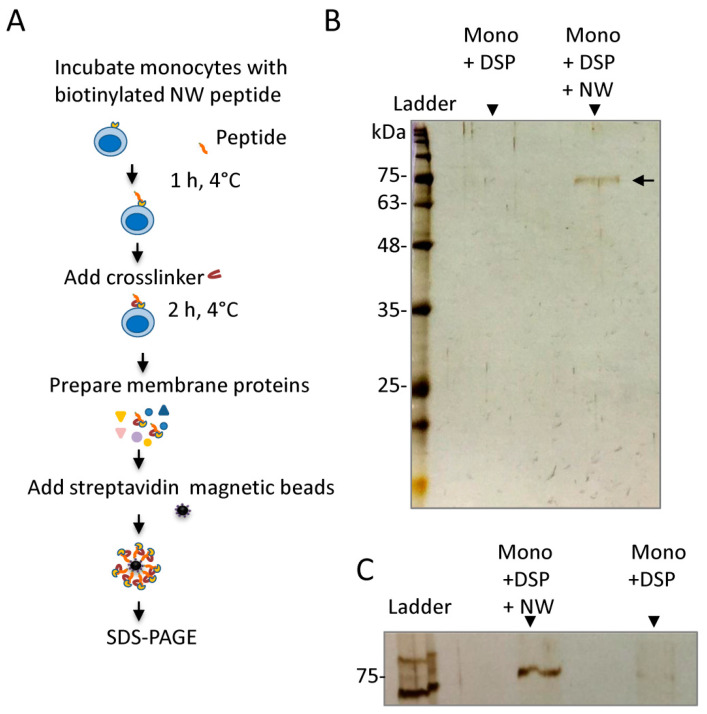
Identification of albumin as a binding partner for the NW peptide. (**A**) Experimental workflow of the chemical cross-linking. In this protocol, monocytes were first incubated with the biotinylated NW peptide and then the chemical cross-linker DSP was added to stabilize the interaction between the NW peptide and its binding receptor(s). After cell lysis, membrane proteins were prepared and streptavidin magnetic beads were applied to pull down the peptide–protein complexes prior to SDS-PAGE analysis. (**B**) Analysis of the eluted proteins by SDS-PAGE. After electrophoresis, the gel was silver stained. As a control, monocytes were incubated with DSP only. A single sharp band was identified in the sample incubated with both the biotinylated NW peptide and the DSP cross-linker. (**C**) An additional experiment. Experimental conditions are as in (**B**). The data shown in (**B**,**C**) are representative of six independent experiments. DSP, Dithiobis(succinimidyl propionate); NW, refers to the peptide’s name; SDS-PAGE, Sodium dodecyl-sulfate polyacrylamide gel electrophoresis.

Using phage display technology, we identified a series of peptides that bind to human monocytes and macrophages. Among the identified peptides, a peptide named NW binds with high affinity to human monocytes and macrophages [22]. To identify the NW binding receptor(s), we first used a chemical cross-linking methodology [23,24]. The benefit of cross-linking on live cells is that the peptide–receptor interaction can be captured in its native environment, which avoids the risk of the receptor losing structure during cell lysis. In these experiments, freshly isolated blood monocytes were incubated with the biotinylated NW peptide, washed to remove unbound peptides, and then incubated with DSP, a chemical cross-linker. Subsequently, membrane proteins were prepared and the biotinylated NW peptide–receptor complexes were captured using streptavidin-conjugated magnetic beads, as illustrated in Figure 1A. Analysis of the captured proteins with SDS-PAGE followed by silver staining identified a unique protein band that was enriched in the sample co-incubated with the NW peptide and DSP (Figure 1B,C). The protein band was cut from the gel and its identity determined by mass spectrometry. Of the identified proteins, human albumin displayed a high probability, with the highest number of exclusive peptides and high sequence coverage.

**Figure 2:**
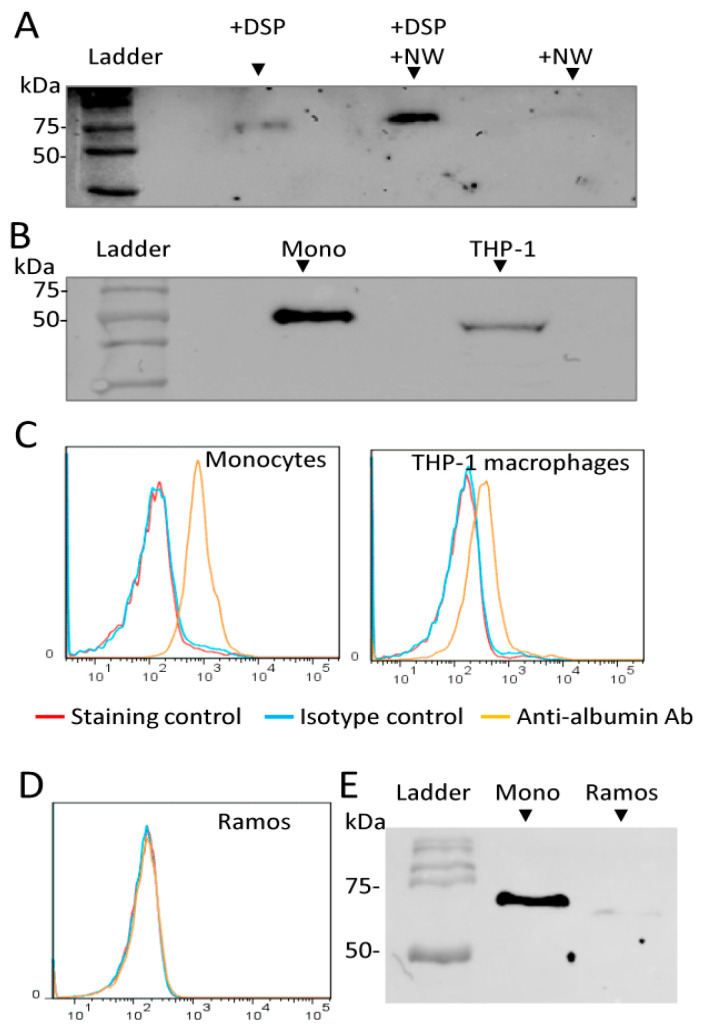
Albumin is present in pulled-down proteins and on the cell surface of monocytes/macrophages. (**A**) After cross-linking, proteins in cell lysates were pulled down by streptavidin magnetic beads and then analyzed by Western blotting using an anti-albumin monoclonal antibody. Lane 1, monocytes incubated with only DSP; Lane 2, monocytes incubated with the NW peptide and DSP; Lane 3, monocytes incubated with only NW peptide. (**B**) Analysis of membrane proteins by Western blotting. Membrane proteins from monocytes and THP-1 macrophages were prepared and analyzed by Western blotting using an anti-albumin monoclonal antibody. (**C**,**D**) Representative histograms showing the analysis of surface albumin by flow cytometry in monocytes, THP-1 macrophages, and Ramos cells. (**E**) Analysis of membrane proteins by Western blotting using an anti-albumin monoclonal antibody. (**A**–**E**) The results presented are representative of at least three independent experiments. DSP, Dithiobis(succinimidyl propionate); Mono, Monocytes; kDa, Kilodaltons.

To validate that the captured protein was albumin, similar experiments were repeated and the proteins were separated by SDS-PAGE and then analyzed by Western blotting using a monoclonal antibody against albumin (Figure 2A). Albumin was detected in the protein sample pulled down with the NW peptide. Similar results were obtained with THP-1 macrophages. The presence of albumin on the cell surface of monocytes and THP-1 macrophages was confirmed by Western blotting and flow cytometry analyses (Figure 2B,C). To further investigate the role of surface albumin in peptide binding, we have used the human Burkitt’s lymphoma cell line, Ramos, as a control that does not bind to the NW peptide. In contrast to THP-1 macrophages and monocytes, Ramos did not display albumin on the cell surface (Figure 2D). In accordance with the flow data, analysis of membrane proteins by Western blotting revealed no significant reactivity in the fraction isolate from Ramos cells when compared to that isolated from blood monocytes (Figure 2E).

### 2.2. Maleic-Anhydride-Modified Albumin Competes with the NW Peptide Binding to Monocytes

**Figure 3:**
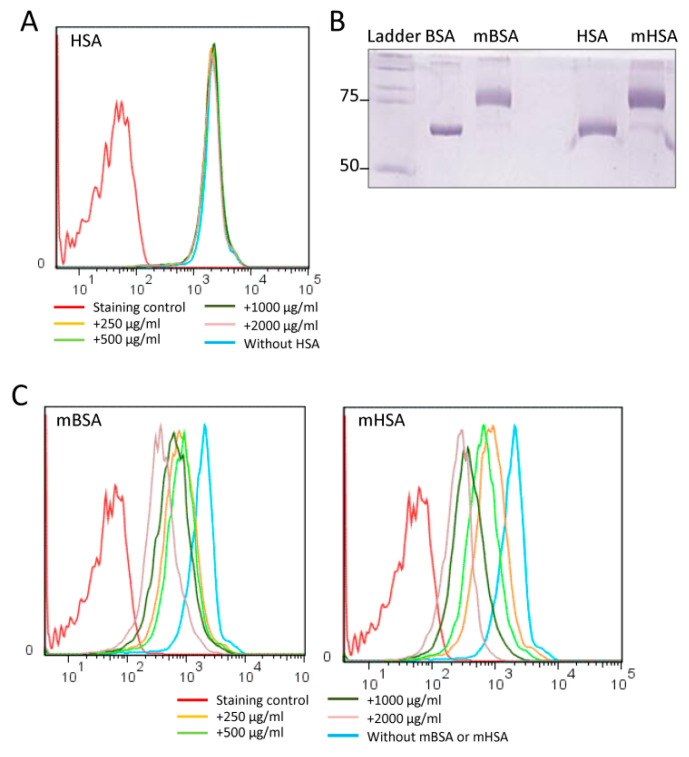
Effects of unmodified and modified albumin on NW peptide binding to monocytes. (**A**) Unmodified albumin does not compete with the NW peptide binding. Monocytes were pre-incubated with various concentrations of unmodified human serum albumin (HSA) prior to the addition of NW peptide, staining, and analysis by flow cytometry. (**B**) Analysis of maleic-anhydride-modified albumin by SDS-PAGE. HSA and bovine serum albumin (BSA) were modified by maleic anhydride as described in the Section 4 and then analyzed by 10% SDS-PAGE followed by Coomassie blue staining. Unmodified HSA and BSA were included as controls. (**C**) Maleic-anhydride-modified albumin competes with the NW peptide binding to monocytes. Monocytes were pre-incubated with various concentrations of modified albumin before being stained with the NW peptide and analyzed by flow cytometry. The data shown in (**A**,**C**) are representative of at least eight independent experiments. m, Modified.

To verify the involvement of albumin in peptide binding to monocytes, we first tested whether unmodified human serum albumin (HSA) would compete with the NW peptide binding to monocytes (Figure 3A). Incubation of the monocytes with HSA did not block the binding, indicating that there is no direct interaction between the NW peptide and unmodified albumin. Comparable results were obtained with bovine serum albumin (data not shown). As shown in Figure 1 and Figure 2, the molecular weight of the captured albumin is around 75 kDa, whereas that of natural albumin is 66 kDa. This observation suggests that the NW-peptide-captured albumin is likely to be modified.

Notably, maleic-anhydride-modified albumin is the most common form of modified albumin within the body. To investigate the potential inhibitory effect of this modification on the NW peptide binding, maleic-anhydride-modified BSA (mBSA) or HSA (mHSA) was prepared and the molecular masses of these modified proteins were characterized by SDS-PAGE (Figure 3B). Under our experimental conditions, amino acids arginines and lysines on both proteins should be fully modified by maleic anhydride (see the Section 4). Their molecular weight reached 75 kDa, which is comparable to the mass of captured albumin. In contrast to unmodified albumin, maleic-anhydride-modified albumin did inhibit the binding of NW peptide to monocytes, but only at high concentrations (Figure 3C). We next performed two kinds of competition experiments with modified albumin. Monocytes were incubated with the modified albumin for 30 min and either washed or not with FACS buffer before the addition of the NW peptide. This washing step is aimed at excluding the interaction between the NW peptide and modified albumin. Under both experimental conditions, modified albumin inhibited the binding of the NW peptide to monocytes (Supplemental Figure S1A,B). Again, higher concentrations were needed to achieve around 50% inhibition effect.

### 2.3. Cell Surface Prohibitins May Function as a Receptor for the NW Peptide

**Figure 4:**
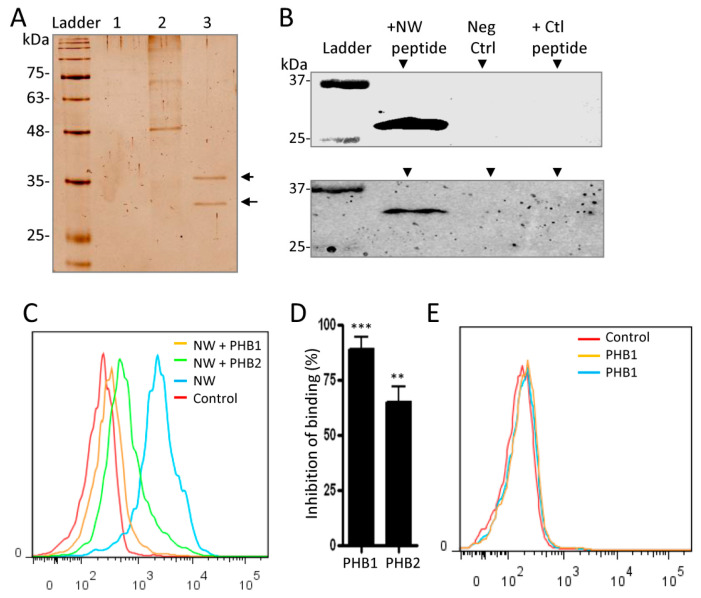
NW peptide binds to prohibitins. (**A**) Proteins in whole-cell protein lysates were incubated with the biotinylated NW peptide or the control peptide, and peptide-binding proteins were pulled down by streptavidin magnetic beads and analyzed by SDS-PAGE followed by silver staining. Protein lysates incubated with only streptavidin-magnetic beads served as an additional control. Lane 1, proteins incubated with beads; Lane 2, proteins incubated with the control peptide; Lane 3, proteins incubated with the NW peptide. Two bands within the range of 35 to 25 kDa were detected in Lane 3. (**B**) Analysis of the pulled-down proteins by Western blotting. Experimental conditions are as in (**A**). Proteins were electrotransferred to nitrocellulose membranes and probed with a monoclonal antibody against PHB1 or PHB2 protein. Neg Ctrl, negative control (no peptide was added to the protein extracts). (**C**) Prohibitins inhibit the binding of the NW peptide to monocytes. The NW peptide (0.010 μg) was pre-incubated or not with prohibitins (3 μg each) for 30 min at RT before being added to monocytes and analyzed by flow cytometry. (**D**) Inhibition of peptide binding by prohibitins. Average values and SD of three independent experiments are shown. (**E**) Prohibitins did not show binding to monocytes. Monocytes incubated or not with prohibitins were washed and incubated with a PE-conjugated anti-His-tag monoclonal antibody to detect prohibitins by flow cytometry analysis. The data are representative of at least three independent experiments. ** *p* < 0.01; *** *p* < 0.005. PHB, Prohibitin; kDa, Kilodaltons.

Given that modified albumin inhibited the binding of the peptide to monocytes at only high concentrations, we asked whether other cell surface proteins may function as a receptor. To investigate this, we used the biotinylated NW peptide as bait in a pull-down assay with whole-cell lysates prepared from human blood monocytes. The biotinylated NW peptide and its interacting proteins were captured using streptavidin-conjugated magnetic beads. After the beads were washed extensively, the captured proteins were eluted and analyzed by 10% SDS-PAGE (Figure 4A). Two unique protein bands were identified in the protein samples eluted from pull-down with the NW peptide (Lane 3), which were absent after pull-down using only beads or control peptide (Lanes 1 and 2, respectively). Notably, these two proteins were also detected in samples prepared from THP-1 macrophages and not in control samples (data not shown). Mass spectrometric analysis identified the upper band as prohibitin 2 (PHB2) and the lower band as prohibitin 1 (PHB1), two conserved 37 and 30 kDa proteins, respectively, that are involved in diverse cellular processes [25,26].

To verify the presence of PHB1 and PHB2 within eluted proteins, identical experiments were repeated and proteins were separated by SDS-PAGE and then analyzed by Western blotting using a monoclonal antibody specific for PHB1 or PHB2. Both proteins were present in the protein samples eluted from pull-down with the NW peptide, but not in the control samples (Figure 4B). Analysis of gene expression by Western blotting showed that the PHB1 protein level was around 0.5-fold higher than that of PHB2 (Supplemental Figure S2A–C). Although this difference in gene expression may in part explain the relative amounts of pulled-down proteins, the NW peptide may display different affinities to PHB1 and PHB2 proteins. To confirm the interaction between the NW peptide and prohibitins, competition experiments using recombinant proteins were performed. Pre-incubation of the peptide with either PHB1 or PHB2 inhibited the binding to monocytes (Figure 4C,D). Under our experimental conditions, PHB1 was more effective than PHB2 in inhibiting the peptide from binding to monocytes. Neither of the proteins bound to monocytes, supporting their direct interaction with the NW peptide (Figure 4E).

### 2.4. Prohibitin 1 Is a Potential Cell Surface Receptor for the NW Peptide on Monocytes

**Figure 5:**
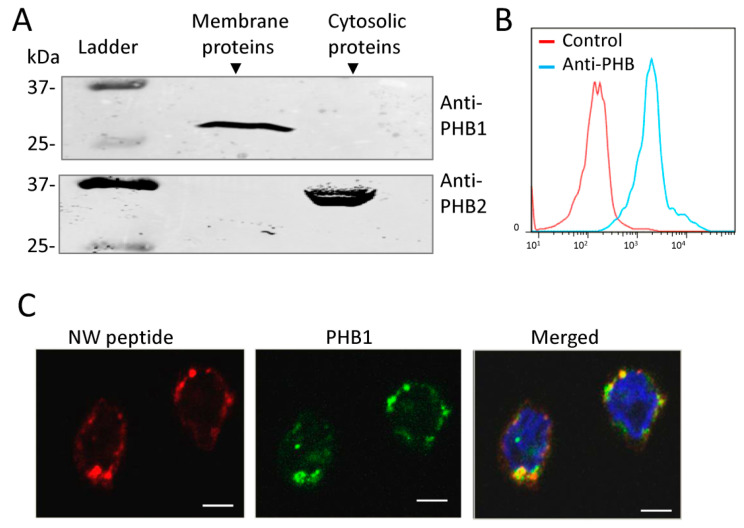
Analysis of prohibitin cellular localization in monocytes. (**A**) Western blot analysis. Membrane and cytosolic proteins were prepared from monocytes and analyzed via Western blotting using monoclonal antibodies against PHB1 or PHB2. (**B**) A representative flow cytometry histogram showing the expression of PHB1 on the surface of monocytes. (**C**) PHB1 co-localizes with the NW peptide on the plasma membrane of monocytes. Monocytes were co-stained with the NW peptide and a mouse anti-prohibitin monoclonal antibody, followed by incubation with PE-streptavidin and anti-mouse IgG-FITC, and then stained with DAPI. Cells were mounted on slides, and confocal images were recorded. The data are representative of at least three independent experiments. Scale bar = 20 μm. PHB, Prohibitin; kDa, Kilodaltons.

Prohibitins are reported to be localized in several subcellular compartments, including the nucleus, mitochondria, and plasma membrane, to carry out their diverse functions [26]. Moreover, PHB1 has been detected in the circulatory system [27]. However, no information is available about the display of prohibitins on the surface of human monocytes. To investigate the possibility of this scenario, cytoplasmic and membrane proteins were prepared from monocytes and analyzed by Western blots using PHB1- or PHB2-specific monoclonal antibodies. The analyses revealed that PHB1 is localized in the membrane fraction while PHB2 is localized in the cytoplasmic fraction (Figure 5A). The purity of the protein fractions was verified using β-actin and the voltage-dependent anion selective channel 1 (VDAC1) as markers for cytoplasm and plasma membrane, respectively. Western blotting showed that β-actin was only present in the cytoplasmic fraction whereas VDAC1 was only present in the membrane fraction (Supplemental Figure S3A,B). These results would indicate that pure cytoplasmic and membrane fractions have been isolated.

We next analyzed the display of PHB1 on the monocyte cell surface using flow cytometry. Freshly isolated blood human monocytes were stained with an anti-PHB monoclonal antibody and then analyzed by flow cytometry. As shown in Figure 5B, most of the cells were stained with the antibody. Thus, PHB1 is located on the monocyte cell membrane. To further support the role of cell surface PHB1 in NW peptide binding to monocytes, we also assessed its membrane localization by immunofluorescence. Consistent with the flow cytometry data, confocal imaging revealed that PHB1 is localized on the plasma membrane of monocytes (Figure 5C). Additionally, it is co-localized with the NW peptide (panel merged). Together, these data suggest that PHB1 can function as the membrane receptor for the NW peptide.

### 2.5. NW Peptide Interacts with Intracellular Prohibitins

**Figure 6:**
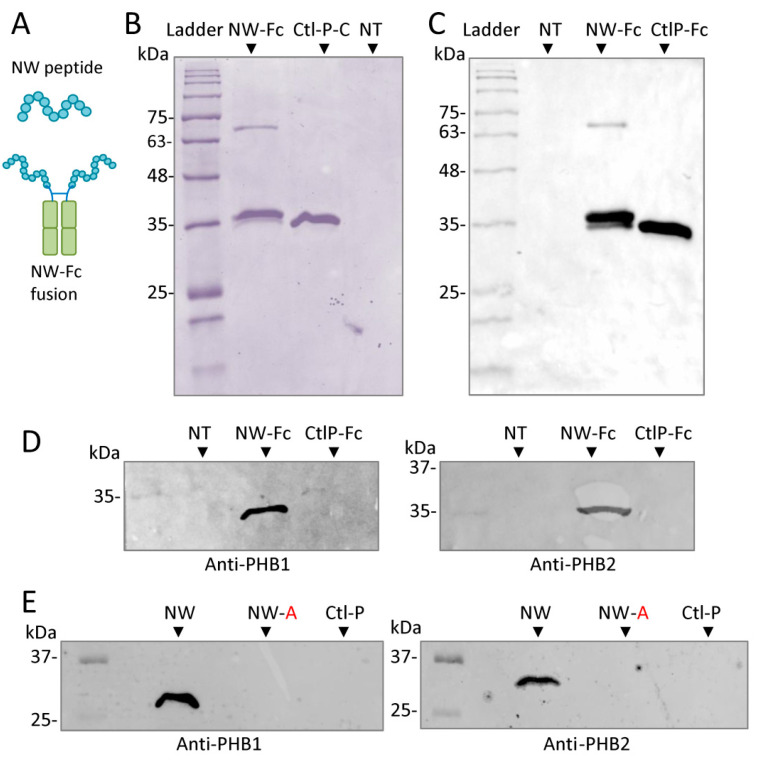
NW peptide interacts with intracellular prohibitins. (**A**) Schematic illustration of the peptide–Fc fusion construct. (**B**) The peptide–Fc fusions (NW–Fc and CtlP–Fc) were expressed in HEK293T cells, purified as described in the Section 4, and then analyzed by SDS-PAGE, followed by Coomassie blue staining. Duplicate gels were transferred to nitrocellulose membranes and probed with either anti-human IgG Fc monoclonal antibody (**C**) or anti-PHB1 or anti-PHB2 monoclonal antibodies (**D**). NT proteins were prepared from non-transfected cells. (**E**) Replacement of W with A at positions 2, 6, and 12 (NW-A) inhibited the binding of the NW peptide to prohibitins. Whole-cell protein lysates were incubated with either the wild-type NW peptide (NWYLPWLGTNDW), the alanine-substituted NW peptide (NAYLPALGTNDA), or the control peptide (FYPSYHSTPQRP) and then processed as described for Figure 4A,B. CtlP, control peptide.

**Figure 7:**
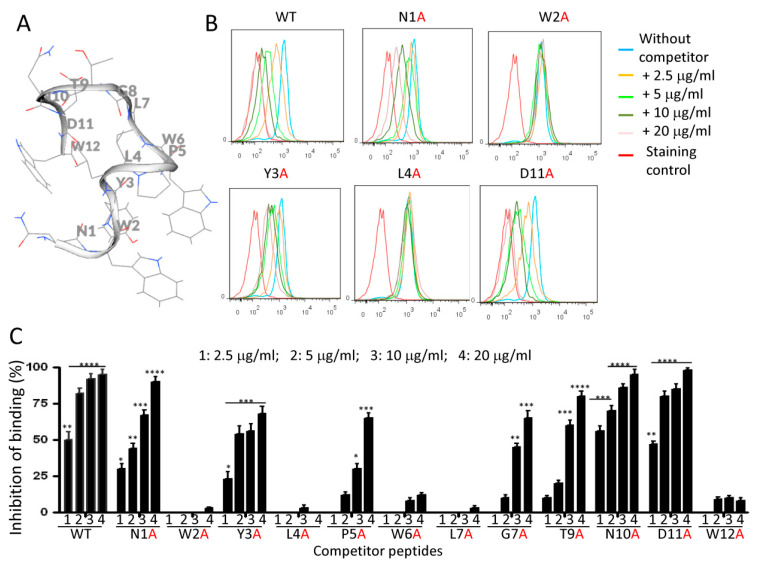
Summary of competition assays using alanine-substituted NW peptide. (**A**) Carton diagram of the NW peptide (side view). Amino acids represented as lines are indicated. (**B**) Representative histograms showing the inhibitory effect of alanine-substituted peptides on the NW peptide (0.05 μg/mL) binding to monocytes. Four concentrations (1–4) of each peptide competitor were tested. (**C**) Percentages of inhibition were calculated from the geometric mean fluorescence intensity. Average mean values and SD of three independent experiments are shown. The numbers 1 to 4 correspond to the used peptide concentrations. * *p* < 0.05, ** *p* < 0.01, *** *p* < 0.005, and **** *p* < 0.0002.

To investigate whether intracellular PHB1 and PHB2 interact directly with the NW peptide, the NW peptide and the control peptide were expressed in HEK293T as fusions with the Fc domain of human IgG to facilitate their purification using protein A/G magnetic beads (Figure 6A,B). The purified fusion proteins were analyzed by Western blotting using anti-human IgG1 Fc antibodies (Figure 6C). The data confirmed the purification of the two peptide–Fc fusion proteins. As shown in Figure 6D, purified NW–Fc, but not control-peptide–Fc fusion (CtlP–Fc), was able to pull down PHB1 and PHB2. Thus, both proteins interact directly with the NW peptide. In support of this finding, recombinant PHB1 and PHB2 proteins inhibited the binding of the NW peptide to monocytes (see Figure 4C,D). Hence, the NW peptide could bind not only to membrane-associated PHB1 but also to intracellular PHB1 and PHB2. Thus, it can be used to modulate the intracellular functions of prohibitins. To assess the role of the amino acid tryptophan (W) in the peptide binding to prohibitins, we analyzed the effects of its replacement at positions 2, 6, and 12 with alanine (A) on peptide binding to PHB1 and PHB2 (Figure 6E). Alanine mutations completely inhibited peptide binding (see also Figure 7).

### 2.6. Effects of Alanine Mutations on Peptide Binding to Monocytes

Alanine-scanning mutagenesis is a widely used strategy for identifying peptide residues that are important for function or ligand binding [28]. This method was used to define the primary structural requirements for NW peptide binding to monocytes. Peptide derivatives were synthesized with 1 of the 12 residues of the NW peptide (N^1^W^2^Y^3^L^4^P^5^W^6^L^7^G^8^T^9^N^10^D^11^W^12^) replaced with alanine (Supplemental Table S1). The peptide derivatives were used to compete with the biotinylated wild-type NW peptide for binding to monocytes, as described in the Section 4. Figure 7A,B shows the putative 3D structure of the NW peptide and some representative examples of cytometric histograms showing the inhibition of the NW binding to monocytes by the alanine-substituted peptides. A summary of the percentages of inhibition of the peptide binding at various competitor concentrations is shown in Figure 7C. As expected, the reference wild-type (WT) peptide (unmodified NW peptide) competed effectively with the biotinylated NW peptide to bind monocytes. The replacement of tryptophan (W) at position 2, 6, or 12 by a single alanine abolished the binding. No significant inhibitory effect was seen even at high competitor peptide concentrations (concentration 4 = 20 μg/mL; 200-fold excess of competitor peptides over the biotinylated NW peptide). Likewise, the replacement of leucine (L) at position 4 or 7 with alanine totally inhibited the peptide binding as no competition with the mutant peptides was found. These results indicate that both W and L are essential for the NW peptide binding to its receptor expressed on the surface of monocytes. In contrast, the replacement of asparagine (N) at position 1 or 10 and aspartic acid (D) at position 11 with a single alanine did not affect the binding of the alanine-substituted peptides. Indeed, these mutant peptides effectively competed with the biotinylated NW peptide to bind monocytes and the percentages of inhibition were comparable to that obtained with the reference peptide (WT) (Figure 7C). Position-specific replacement of the other amino acids by alanine reduced but did not eliminate peptide binding.

**Figure 8:**
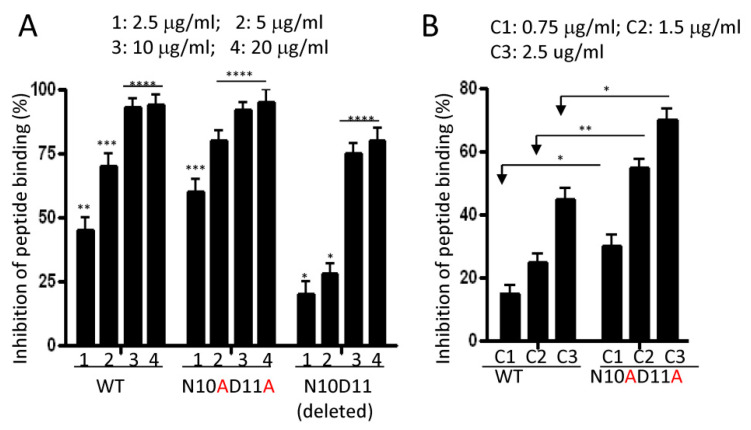
Competition assays. (**A**) Competition using peptides with N10D11-modified positions. (**B**) Comparison of the effectiveness of competitor peptides in inhibiting the binding of the biotinylated NW peptide to monocytes. Different concentrations of competitors were used. Experimental conditions are as in Figure 7. Percentages of inhibition were calculated from the geometric mean fluorescence intensity. Average mean values and SD of three independent experiments are shown. * *p* < 0.05, ** *p* < 0.01, *** *p* < 0.001, and **** *p* < 0.0002.

The finding that the mutant peptides still exhibit a high affinity as the wild-type peptide demonstrates that the amino acids N1, N10, and D11 are not essential for the NW peptide binding. Similar to the single alanine mutation alone, the N10AD11A double mutant effectively inhibited the binding of the NW peptide to monocytes (Figure 8A). However, the deletion of N10D11 significantly reduced the binding, as seen by the approximately 1- to 2-fold reduction in the percentage of inhibition as compared to the reference peptide (WT) at concentrations 1 and 2, respectively (*p* < 0.01). These results would suggest that there is a space requirement between threonine 9 (T9) and W12. As shown in Figure 7A, the peptide forms a β–turn at the N10 position to bring W12 near W2 for possible interaction with the receptor. Turns generally occur when the protein/peptide chain needs to change direction in order to connect two other elements of secondary structures [29]. Previous studies have shown asparagine, aspartic acid, glycine, and proline to be the most common residues forming β–turns [29]. During this study, we noted that the N10AD11A mutations increased the affinity of the mutant peptide to monocytes. As illustrated in Figure 8B, this mutant peptide competed for binding to monocytes much better than the reference WT peptide (*p* < 0.05). Hence, this substitution not only seems to preserve the active structure of the peptide but also may facilitate docking to the receptor(s).

**Figure 9:**
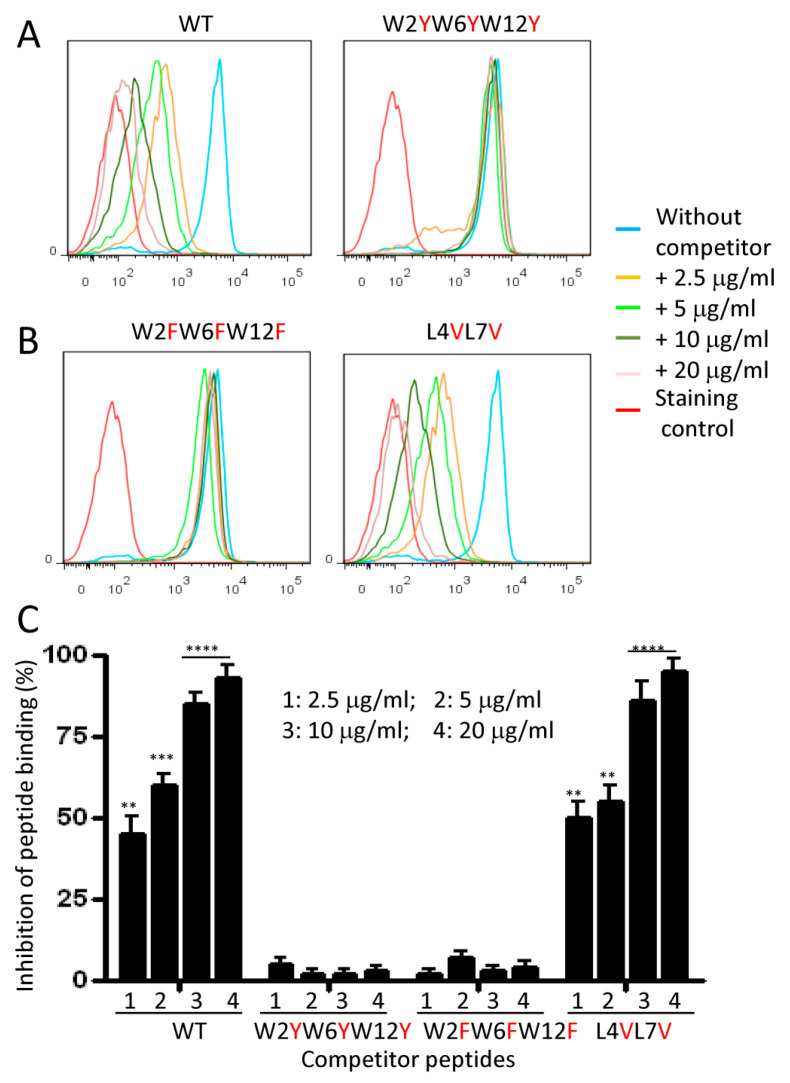
Competition assays using peptides with the neutral replacement of W or L. (**A**,**B**) Representative histograms showing the inhibitory effects of tyrosine—(Y)-, phenylalanine—(F)-, or valine—(V)-substituted peptides on the biotinylated NW peptide binding to monocytes. Experimental conditions are as in Figure 7. The reference NW peptide (WT) was included as a positive control. (**C**) Percentages of inhibition were calculated from the geometric mean fluorescence intensity. Average mean values and SD of three independent experiments are shown. ** *p* < 0.01, *** *p* < 0.001, and **** *p* < 0.0002.

To further understand the requirement of W and L in the NW peptide binding to monocytes, we analyzed the effects of their neutral replacement on binding (Figure 9A–C). The replacement of W by either tyrosine (Y) or phenylalanine (F) did not inhibit the binding of the WT peptide even at high concentrations. This result implies that the side chain of tryptophan (indole ring), which is aromatic with a binuclear structure, is indispensable for the NW peptide to bind monocytes. In contrast, the neutral replacement of L with valine (V) did not affect peptide binding and competition with the biotinylated NW peptide to bind monocytes (Figure 9B,C). Hence, L can be replaced with V 

## 3. Discussion

Unlike conventional antibodies, peptides are relatively small and hence easy to synthesize and modify [30]. Their small size allows their diffusion into tumor tissues and enables them to reach deep into the tumor microenvironment. Thus, peptides capable of homing in onto tumors could function as effective drug-delivery agents, overcoming the obstacles with using antibodies, which have difficulties in reaching the interior of large tumor masses with limited blood supply [31]. The use of random peptide phage libraries led to selections of hundreds of peptides against target cells, both normal and cancer cells [21,32]. However, the receptors they bind to have only been identified for a few peptides, such as those targeting tumor-associated integrins [33,34]. In this study, membrane-associated PHB1 and PHB2 were identified as potential receptors for the NW peptide. Their localization in the monocyte/macrophage plasma membrane suggests that they may have important biological roles that have yet to be elucidated.

To fully unravel the contribution of each amino acid for the NW peptide binding to monocytes, alanine-scanning mutagenesis was performed. Our results demonstrate that W and L play a major role in the interaction of the peptide with monocytes. Being hydrophobic, W and L are important contributors for anchoring membrane proteins within the cell membrane and both are involved in protein–protein interactions [35]. Additionally, it appears that the side chain of tryptophan (indole ring) is indispensable for peptide binding since it cannot be replaced with either tyrosine or phenylalanine. Out of the 12 residues, only 3 amino acids, N1, N10, and D11, can be exchanged with alanine without compromising peptide binding. On comparing the inhibitory effects of mutant peptides to that of the wild-type peptide, the replacement of N10D11 with alanine seems to increase the binding of the alanine-substituted peptide to monocytes (Figure 8B). In future studies, we will take these findings into consideration for the design of peptides with various affinities.

To identify the peptide-binding receptor(s), we used two different strategies, namely peptide cross-linking to live cells by the DSP cross-linker and pull-down assays with the biotinylated NW peptide. Western blot analysis of eluted proteins following cross-linking identified albumin as a binding partner. Competition experiments with maleic-anhydride-modified albumin suggest that there is a possible interaction with the NW peptide on the surface of monocytes. It should be noted that the reactivity of the cross-linker is non-selective toward the binding receptor (s), allowing any protein near the peptide free amine groups (-NH_2_) to be cross-linked. The NW peptide has only one free amino group at the N-terminus and thus may reduce the probability of capturing the real receptor(s).

In contrast to cross-linking results, the pull-down experiments revealed prohibitins as binding partners. Moreover, Western blot analysis using either PHB1- or PHB2-specific antibodies confirmed the presence of prohibitins in the analyzed samples. Additionally, the NW peptide showed co-localization with PHB1 on the membrane of monocytes. The data from the NW–Fc fusion protein experiments indicate that the NW peptide directly interacts with intracellular PHB1 and PHB2 proteins. In support of this conclusion, purified recombinant PHB1 and PHB2 proteins bound to the NW peptide and inhibited its binding to monocytes. Unlike the WT peptide, the mutant peptide harboring A at positions 2, 6, and 12 instead of W did not interact with PHB1 or PHB2. This means that W is indispensable for the peptide to bind prohibitins.

Some forms of PHB1 proteins have been reported to interact with low-density detergent-insoluble lipid raft domains in the plasma membrane, functioning as transmembrane adapters to activate downstream signals [25]. Additionally, cell surface PHB1 and PHB2 were found to be associated with the T cell receptor complex on activated T cells as well as with IgM receptor on B lymphocytes [36,37]. Yurugi et al. found that the expression level of PHB1 on the cell surface was significantly upregulated when T cells were activated [36]. However, the NW peptide did not bind to either resting or activated T cells at the same concentrations that displayed strong binding to monocytes (data not shown). These results raise an important question: Why does the NW peptide bind to monocytes but not to activated T cells that display surface PHBs? Unknown post-translational modifications of PHBs may be required for the NW peptide to bind. Another possible explanation is the existence of cell-type-specific PHBs, as discussed by Kolonin and colleagues, who identified a peptide that only binds PHB2 associated with adipose tissues [38]. Similarly, Sharma and Qadri showed that the Vi polysaccharide of *Salmonella typhi* binds only to prohibitins on intestinal epithelial cells [39]. A similar observation was made for U937 cells that express surface PHB1 and PHB2, but Chikungunya virus that targets cell surface PHB1 and PHB2 did not bind to U937 cells [40,41]. Therefore, additional membrane proteins or the rearrangement of prohibitins within the cell membrane may define the specificity of prohibitins. Unlike intracellular prohibitins, some binding sites may be hidden when they are located on the cell membrane. The binding of the NW peptide to a potential maleic-anhydride-modified albumin receptor, yet to be identified, may facilitate the binding of the NW peptide to surface-associated prohibitins. The maleic anhydride modification confers to albumin a negatively charged surface under the physiological pH conditions. This charge may have an effect on the cell surface of monocytes, facilitating the exposure of cell surface prohibitins or binding sites. In this respect, the incubation of peripheral blood mononuclear cells with maleic-anhydride-modified albumin conferred NW peptide binding to the lymphocyte population (Supplemental Figure S4).

**Figure 10:**
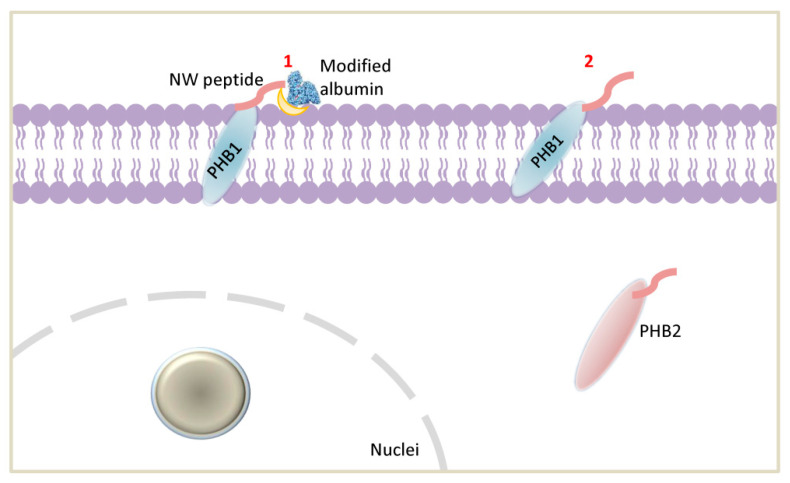
A schematic representation of the proposed mechanisms of NW peptide binding to monocytes. In model 1, NW peptide binds to surface-displayed PHB1 with the help of a modified albumin receptor. In model 2, the NW peptide binds directly to PHB1 on the cell surface of human monocytes. The binding mode somehow resembles the mechanism used by some viruses to enter human cells (see discussion). PHB1, Prohibitin 1; NW refers to the peptide’s name.

It has been reported that certain viruses exhibit a more complex receptor dependency that involves binding with at least two diverse plasma membrane receptors/proteins. The first receptor allows the virus to attach to the cell surface rapidly, while the second receptor facilitates the virus entry into the intracellular environment, e.g., HIV1, HCV, HSV-1 and -2, adenovirus, and measles virus [42,43,44,45]. Additionally, it has been showed that PHB1 and PHB2 mediate the entry of HCV virus, Dengue-3 virus, Chikungunya virus, and HIV virus into host cells [41,43,46,47]. Based on these observations and the present data, we propose two potential mechanisms for how the NW peptide binds target cells (Figure 10): (1) a model where the binding of the peptide to PHB1 on monocytes or macrophages is facilitated by a potential modified albumin receptor or other factors yet to be identified and (2) a model where the peptide binding only involves surface PHB1. Future studies using PHB1 and PHB2 knockout cells will clarify their roles in NW peptide binding and internalization.

## 4. Materials and Methods

### 4.1. Monocyte Purification

Monocytes were isolated from peripheral blood mononuclear cells (PBMCs) using plastic adherence as previously described [22]. Briefly, PBMCs were purified from healthy donor buffy coats by density gradient centrifugation using Lymphoprep™ (Stemcell™ technologies, Cambridge, UK, Cat#07851) according to the manufacturer’s instructions. The isolated PBMCs were washed twice in PBS supplemented with 2% FBS and resuspended in RPMI 1640 medium supplemented with 10% fetal bovine serum and antibiotics (penicillin and streptomycin). The cells were then split into T75 culture flasks (~50 million cells/flask) and incubated at 37 °C for 1–2 h. After incubation, non-adherent cells were removed and adherent cells were washed with PBS or serum-free RPMI 1640 medium. Monocytes were harvested by gentle scraping. In some cases, T cells, B cells, and NK cells were depleted using antibody-conjugated magnetic beads in order to obtain pure monocyte populations. Healthy donor buffy coats were obtained from the blood bank at Ullevål hospital, Oslo, Norway (project code: F8).

### 4.2. Cross-Linking and Pull-Down Assays

We used a cross-linking method to capture the peptide-binding receptor as described by Wu and colleagues [27]. Briefly, monocytes were washed with ice-cold PBS supplemented with 1% BSA and then around 15 × 10^6^ cells were incubated with the biotinylated NW peptide (30 μg) at 4 °C for 1 h with constant agitation. After incubation, cells were washed in ice-cold PBS to remove unbound peptide molecules, followed by incubation with dithiobis(succinimidyl propionate), or DSP, (ThermoFisher Scientific, Oslo, Norway, Cat#22585) at a final concentration of 2 mM at 4 °C for 2 h. The reaction was quenched by the addition of 20 mM Tris-HCl (pH 7.5), and the membrane proteins were prepared using the Mem-PER^TM^ Plus Membrane Protein Extraction Kit (ThermoFisher Scientific, Cat#89842) according to the manufacturer’s instructions. The protein complexes were pulled down using Dynabeads™ Myone™ Streptavidin T1 beads (Invitrogen Dynal As, Oslo, Norway, Cat#65601). Subsequently, the beads were washed extensively in washing buffer (50 mM Tris-HCl, 150 mM NaCl, and 1% NP-40; pH 7.4) to remove unspecific binders. The pulled-down proteins were then eluted from the beads in the presence of 50 mM DTT at 37 °C for 30 min followed by 5 min at 95 °C. Finally, the eluted proteins were separated by SDS-PAGE and visualized by either silver staining or Coomassie blue staining.

For pull-down experiments, total protein lysates were prepared from monocytes (15 × 10^6^ cells). The cells were lysed in cell lysis buffer (50 mM Tris-HCl, 150 mM NaCl, 1% NP-40, 0.05% Tween-20, 6 mg/mL octyl-β-glucoside, and 1:100 protease inhibitor) with constant rotation at 4 °C for 40 min. The samples were vortexed every 10 min. After incubation, the samples were centrifuged at 16,000× *g* at 4 °C for 15 min and the supernatants (300–500 μg) were incubated with either the biotinylated NW peptide or the control peptide (25 μg each) at 4 °C for 1 h with constant rotation. Peptide–protein complexes were subsequently captured using magnetic-streptavidin-conjugated Dynabeads (Invitrogen™, Cat#65601). The beads were washed seven times in washing buffer (50 mM Tris-HCl, 150 mM NaCl, and 1% NP-40; pH 7.4), and then peptide-binding proteins were eluted from the beads with 1:1 PBS and 3xSDS-PAGE loading buffer supplemented with DTT for 30 min at 37 °C followed by 5 min at 95 °C. Finally, eluted proteins were analyzed by SDS-PAGE and visualized by either silver or Coomassie blue staining.

### 4.3. Analysis of the Captured Proteins by Western Blotting

After protein elution, equivalent amounts of samples were analyzed by 10% SDS-PAGE. The proteins were then electrotransferred onto nitrocellulose membranes, blocked with TBS-T buffer (20 mM Tris-HCl, 150 mM NaCl, and 0.1% Tween-20; pH 7.4) supplemented with 5% non-fat milk or BSA at room temperature, and left for 1 h. The membranes were incubated with primary antibodies diluted in TBS-T supplemented with 1% non-fat milk/BSA at 4 °C overnight. The next day, the membranes were washed with TBS-T three times (10 min/wash) prior to incubation with HRP-conjugated secondary antibodies diluted in TBS-T supplemented with 1% non-fat milk/BSA at 4 °C for 1 h. After three washes with TBS-T, antibody binding was detected using the Clarity Western ECL blotting substrate (BioRad, Irvine, CA, USA, Cat#1705061) according to the manufacturer’s instructions. The membranes were visualized with the ChemicDoc™ MP Imaging System (BioRad, Irvine, CA, USA). The antibodies used in this study are indicated in Supplemental Tables S2 and S3.

### 4.4. Chemical Modification of Albumin with Anhydride

Anhydride-modified proteins were produced as previously described [48,49]. Briefly, bovine serum albumin (BSA) or human serum albumin (HSA) was dissolved in 0.1 M phosphate buffer (pH 8.6) to obtain a final concentration of 20 mg/mL. Maleic anhydride (Aldrich^®^, Rockville MD, USA, Cat#M188-25G-A) was dissolved in DMSO to obtain a final concentration of 1 M. Subsequently, the protein solutions were mixed with maleic anhydride to a final concentration of 60 mM by the addition of five equivalent aliquots at 20 min intervals. After each addition, pH was adjusted to 9.0 using 5 M NaOH. The mixtures were incubated at 25 °C for an additional 2.5 h and then dialyzed against PBS overnight with constant gentle stirring. Protein concentrations were determined via Bradford assay.

### 4.5. Analysis of Albumin Effects on NW Peptide Binding to Monocytes

To examine the inhibition of the NW peptide binding to monocytes by maleic-anhydride-modified albumin, we used flow cytometry. Briefly, monocytes were washed twice in FACS buffer (PBS supplemented with 0.5% BSA and 3 mM EDTA) and resuspended to a density of 3–5 × 10^5^ cells/100 µL per sample. The cells were then incubated with different concentrations of unmodified (0.8–2.9 mg/mL) or modified BSA (1–3.8 mg/mL) for 40 min at 4 °C. After incubation, the biotinylated NW peptide (0.1–0.5 µg/mL) was added either directly to the samples or subsequent to three washes in FACS buffer. Similarly, the cells were incubated with unmodified or modified HSA and processed, as above. All samples were incubated with the biotinylated NW peptide for 30 min at 4 °C, followed by staining for 30 min at 4 °C with phycoethrin (PE)-conjugated streptavidin (BD Pharmingen™, San Diego, CA, USA) diluted 1:300 in FACS buffer. The cells were washed twice between two incubation steps prior to analysis on a BD FACS Canto II equipped with the BD FACSDiva™ software (BD Biosciences, San Jose, CA, USA). All data were analyzed using FlowJo version 7.6.1 (FlowJo LLC, Ashland, OR, USA).

### 4.6. Analysis of the Prohibition Effects on NW Peptide Binding to Monocytes

To examine the effect of recombinant prohibitins on NW peptide binding to monocytes, the biotinylated NW peptide (0.010 μg) was incubated with either PHB1 or PHB2 (3 μg) at RT for 30 min before being added to monocytes. After 30 min incubation at 4 °C, the cells were washed twice in FACS buffer prior to incubation with PE-conjugated streptavidin (diluted 1:300 in FACS buffer) for 30 min at 4 °C, followed by washing and flow cytometric analysis. The peptide and protein concentrations were determined in preliminary experiments. Recombinant PHB1 (Cat#1381) and PHB2 (Cat#Pro-1533) were purchased from ProSpec-Tany TechnoGene LTD, Ness-Ziona, Israel.

### 4.7. Confocal Microscopy Analysis

Purified monocytes were co-stained with the biotinylated NW peptide in combination with mouse anti-prohibitin monoclonal antibody in Eppendorf tubes. After incubation at 4 °C for 40 min, the cells were washed with FACS buffer and then incubated with PE-conjugated streptavidin and FITC-conjugated anti-mouse IgG for 30 min at 4 °C before being washed and resuspended in 50 μL of PBS buffer. A 20 μL drop of each sample was placed in the center of a glass slide and incubated at RT for 15 min. Thereafter, the samples were fixed with 4% paraformaldehyde, left for 20 min at RT, and washed with PBS. The slides were mounted with DAKO mounting medium and covered by cover slips for imaging with the Zeiss LSM 710 confocal microscope using the Plan-Apochromat 63x/1.40 oil objective (Carl Zeiss Microscopy GmbH, Oberkochen, Germany). Images were processed with the ZEN lite software (Carl Zeiss Microscopy GmbH, version 3.3).

### 4.8. Cloning and Expression of Peptide-Fc Fusions

Overlapping oligonucleotides encoding the NW peptide (NWYLPWLGTNDW) or the control peptide (CPIEDRPMC) with overhanging EcoR1 and BglII restriction sites were made and HPLC-purified by Eurofins Genomics (Germany). After annealing, the minigenes were cloned into pFUSE-hIgG1-Fc2 in frame with the Fc domain of human IgG1 as described previously [50]. Positive clones were verified by restriction mapping and DNA sequencing. Plasmid DNA was isolated from single positive clones using the NucleoSpin plasmid purification kit according to the manufacturer‘s instructions (Macherey-Ngel Inc., Düren, Germany). The following oligonucleotides were used: 

NW peptide

Oigo-1: 5′-aattcgaattggtatctgccttggctggggacgaatgattggggcggca-3′

Oligo 2: 5′-gatctgccgccccaatcattcgtccccagccaaggcagataccaattcg-3′

Control peptide (CP)

Oligo-1: 5′-aattcggcatgccctattgaagacaggcctatgtgcggcggca-3′

Oligo-2: 5′-gatctgccgccgcacataggcctgtcttcaatagggcatgccg-3′

### 4.9. Pull-Down Assays Using Peptide–Fc Fusions

HEK293T cells were seeded in T75 flasks and cultured until they reached 70–80% confluence. The cells were transfected with plasmids encoding NW–Fc or CP–Fc fusion proteins for 48 h prior to harvest. After the cells were washed twice in PBS, they were incubated in lysis buffer (50 mM Tris-HCl, 150 mM NaCl, 1% NP-40, and 1:100 protease inhibitor cocktail; pH 7.4) for 40 min on ice with vortexing every 10 min. Subsequently, the samples were spun down at 16,000× *g* at 4 °C for 15 min and the supernatants containing the extracted proteins were collected. The protein complexes were pulled down using Dynabeads™ Protein G beads (Invitrogen Dynal AS, Oslo, Norway, Cat#10003D). The beads were extensively washed in washing buffer (50 mM Tris-HCl, 150 mM NaCl, and 1% NP-40; pH 7.4) to remove unspecific binders. The pulled-down proteins were then eluted in 50 µL elution buffer (0.1 M citrate; pH 2.5) at RT for 10 min with constant agitation and immediately neutralized with 10 μL of neutralization buffer (1 M Tris-HCl; pH 8.5). Finally, the eluted proteins were separated by 10% SDS-PAGE followed by Coomassie staining or Western blotting.

### 4.10. Analine-Scanning Mutagenesis

Alanine-scanning mutagenesis was used to explore the function of specific amino acid residues on peptide binding to monocytes. A total of 12 mutant peptides were generated where single residues within the sequence NWYLPWLGTNDW were replaced with alanine. Various concentrations (2.5–20 μg/mL) of these mutant peptides were used to compete with the wild-type peptide (0.05 μg/mL) for binding to monocytes using flow cytometry. Four different concentrations were tested for each peptide. We used the geometric mean fluorescence intensity index method to measure the inhibition of peptide binding. The peptides used in this study were synthesized by GeneCust (Dudelance, Luxembourg [51]). The identity of the peptides was confirmed by mass spectrometry and the purity determined by reversed-phase HPLC. The purity was at least 85%.

### 4.11. Statistical Analysis

The data are expressed as the mean based on a minimum of three experiments unless otherwise indicated. Statistical analyses were carried out with GraphPad Prism version 4 (GraphPad Software, San Diego, CA, USA). Statistical significance was evaluated with Student’s *t*-test or a non-parametric ANOVA test. All *p*-values < 0.05 were considered statistically significant.

## 5. Conclusions

Macrophages are a major component of solid cancers and can promote tumorigenesis by facilitating angiogenesis, immunosuppression, and metastasis. Hence, it is of great need to develop delivery agents that target macrophages and their progenitor monocytes. Here, we identified cell surface prohibitins as the receptor for the NW peptide that binds to monocytes/macrophages with high affinity. Additionally, alanine-scanning mutagenesis identified amino acids W and L as essential for the peptide to bind target cells. The data also suggest that the indole ring is the most important moiety in the binding of the W to the receptor. Collectively, the data offer the promise of designing improved targeted therapies to modulate the function of monocytes/macrophages.

## Data Availability

Data supporting the described results are contained within the article and its Supplementary Materials. Public data sources are listed in the Section 4.

## Supplementary Materials

The following supporting information can be downloaded at: https://www.mdpi.com/article/10.3390/ijms23084282/s1.

## Abbreviations

A	Alanine
BSA	Bovine serum albumin
CtlP–Fc	Control-peptide–Fc fusion
DSP	Dithiobis(succinimidyl propionate)
D	Aspartic acid
F	Phenylalanine
HPLC	High-performance liquid chromatography
HSA	Human serum albumin
L	Leucine
mBSA	Maleic-anhydride-modified bovine serum albumin
mHSA	Maleic-anhydride-modified human serum albumin
NT	Non-transfected
PBMCs	Peripheral mononuclear cells
PHB	Prohibitin
SDS-PAGE	Sodium dodecyl-sulfate polyacrylamide gel electrophoresis
TAMs	Tumor-associated macrophages
TME	Tumor microenvironment
VDAC1	Voltage-dependent anion selective channel 1
V	Valine
W	Tryptophan
WT	Wild type
Y	Tyrosine
