# Supplementary material for: Structural Requirements for the Binding of a Peptide to Prohibitins on the Cell Surface of Monocytes/Macrophages
Source: Int J Mol Sci. 2022 Apr 13;23(8):4282. doi: 10.3390/ijms23084282 (PMC9029656; doi:10.3390/ijms23084282)
Supplement: Supplementary file 1 [file ijms-23-04282-s001.zip › ijms-1596383-supplementary.pdf]

Supplementary Materials

Qindong Zhang, Anniken Olberg and Mouldy Sioud

Structural requirement for the binding of a peptide to prohibitins on the cell surface of monocytes/macrophahes. *Int. J. Mol. Sci* 2022,**23**.

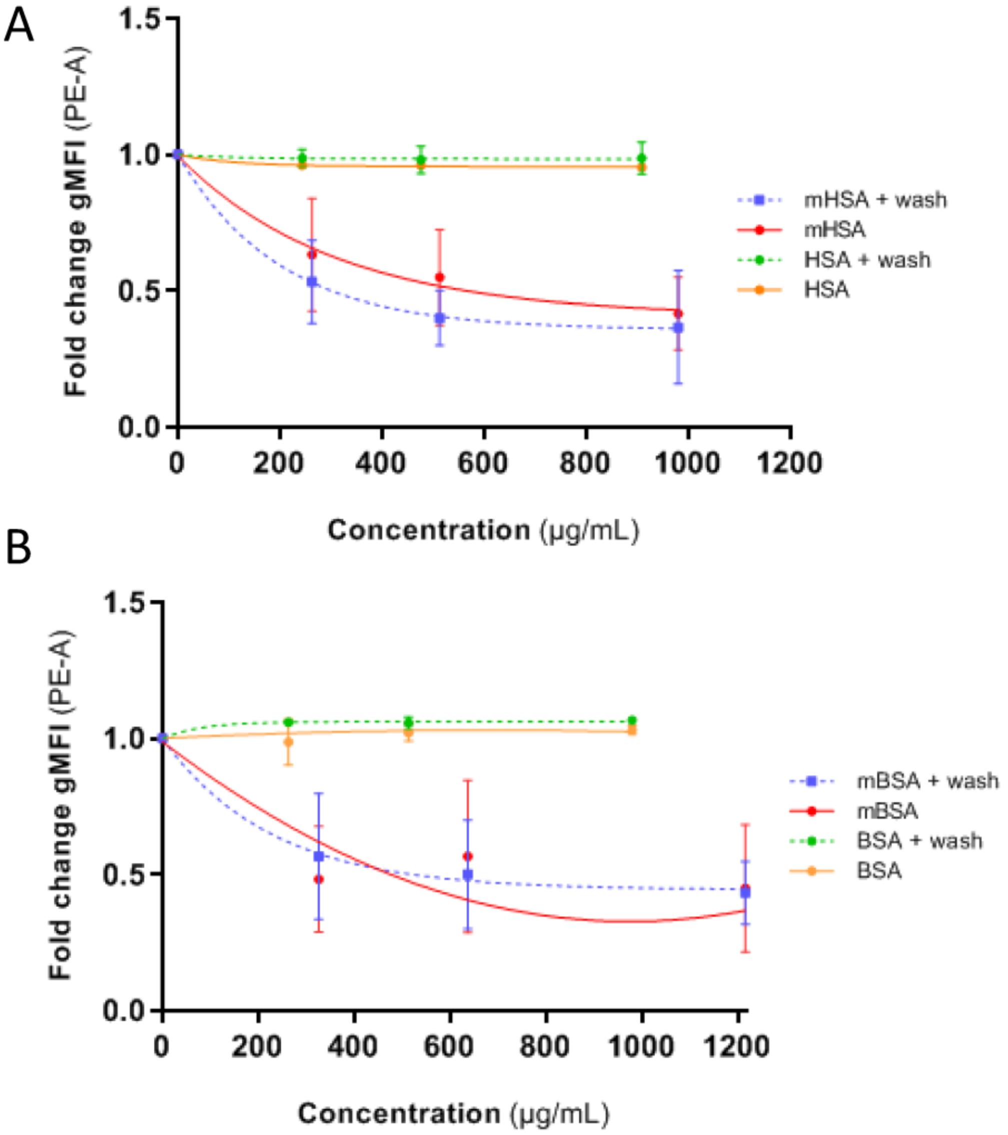

Figure S1. A summary of the competition assays with unmodified and maleic anhydride modified-albumin. Briefly, monocytes were incubated with unmodified or modified (m) HSA (A) for 30 min and washed or not prior to addition of biotinylated NW peptide. After incubation at 4° for 60 min, the cells were washed, stained with PE-streptavidin and then analyzed by flow cytometry. In (B), the cells were incubated with unmodified or modified BSA and processed as in A. Fold changes were calculated from geometric mean fluorescence intensity (gMFI). Average mean values and SD of 3 independent experiments are shown.

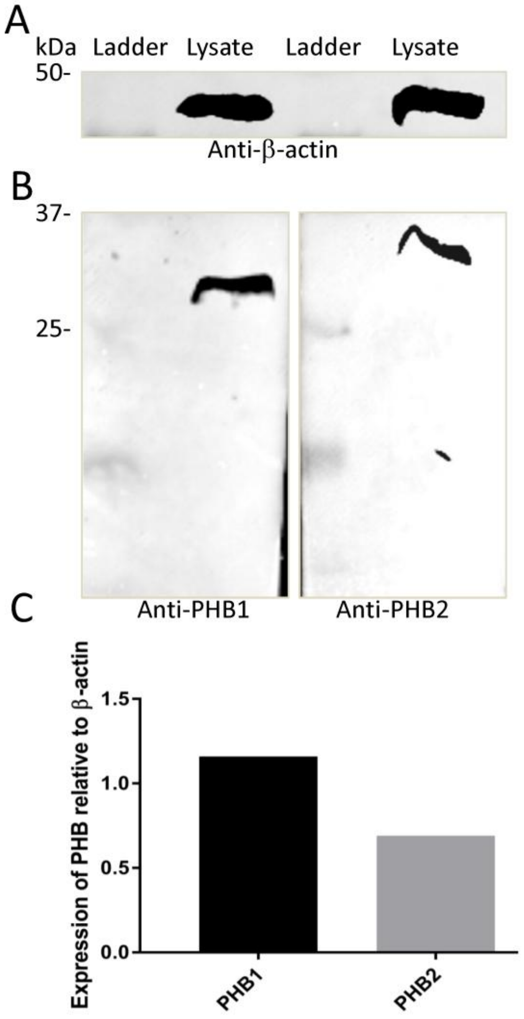

Figure S2. A representative western blot showing the expression of PHB proteins. Whole protein lysate was prepared from freshly isolated blood monocytes as described in Materials and Methods and then the expression of PHB1 and PHB2 (panel **B**) was analyzed by Western blotting with the indicated monoclonal antibodies. To control for protein loading, the upper part of the membrane was probed with an anti β-actin monoclonal antibody (panel **A**). The intensity of each bands was quantified with Image Lab 6.1 software. The ratio of the relative level of PHB1 and PHB2 to β-actin is shown in panel **C**.

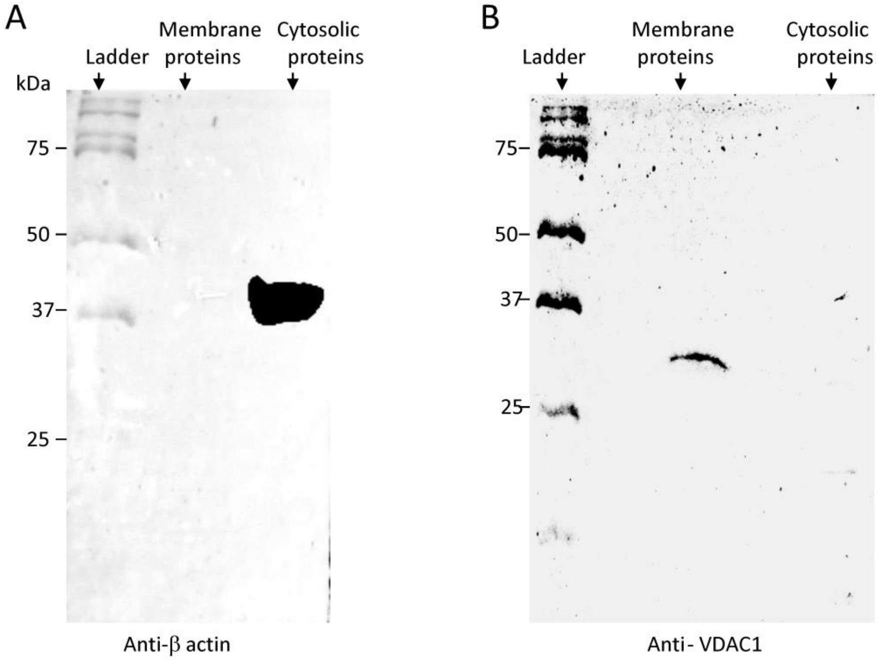

Figure S3. Purity analysis of the membrane and cytosolic protein fractions. Protein fractions were prepared from freshly isolated blood monocytes as described in Materials and Methods and then analyzed by Western blots for the presence of β-actin (**A**) and voltage-dependent anion selective channel 1 (VDAC1) (**B**) using specific monoclonal antibodies. As expected, β-actin was only detected in the cytoplasmic fraction, whereas VDAC1 was detected only in the membrane fraction. Thus, pure cytoplasmic and membrane fractions have been isolated.

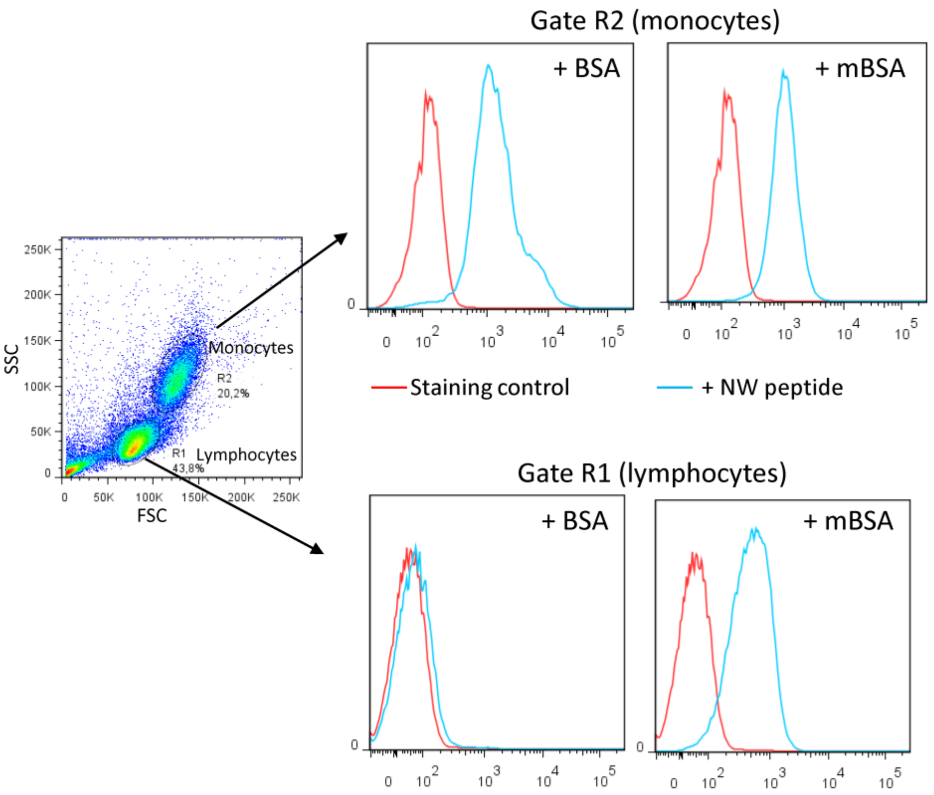

Figure S4. Treatment of peripheral mononuclear cells (PBMCs) with maleic anhydride modified-albumin conferred binding of the NW peptide to the lymphocyte population. PBMCs were incubated with approximately 1 mg/ml unmodified BSA or modified BSA (mBSA) for 30 minutes, prior to addition of biotinylated NW peptide (0.4 µg/µl). The cells were then washed, stained with PE-streptavidin and analyzed by flow cytometry. The fluorescent intensity of gated cell populations under different treatment conditions is indicated. The data are representative of 4 independent experiments.

.

Table S1. Peptide sequences

Peptide	Sequence	Purity%	m/Z
NW	NWYLPWLGTNDW	97.9	783.20
NW-N1A	**A**WYLPWLGTNDW	92.53	767.16
NW-W2A	N**A**YLPWLGTNDW	96.69	788.22
NW-Y3A	NW**A**LPWLGTNDW	96.37	790.32
NW-L4A	NWY**A**PWLGTNDW	96.07	800.51
NW-P5A	NWYL**A**WLGTNDW	94.39	799.25
NW-W6A	NWYLP**A**LGTNDW	85.2	812.22
NW-L7A	NWYLPW**A**GTNDW	97.1	815.45
NW-G8A	NWYLPWL**A**TNDW	95.13	799.51
NW-T9A	NWYLPWLG**A**NDW	95.47	815.33
NW-N10A	NWYLPWLGT**A**DW	85.76	850.61
NW-D11A	NWYLPWLGTN**A**W	87.95	799.65
NW-W12A	NWYLPWLGTND**A**	92.31	814.34
NW-FFF	N**F**YLP**F**LGTND**F**	86.13	724.80
NW-YYY	N**Y**YLP**Y**LGTND**Y**	98.19	749.00
NW-VV	NWY**V**PW**V**GTNDW	87.37	766.79
NW-AA	NWYLPWLGT**AA**W	90.21	1479.25
NW-10	NWYLPWLGTW	95.72	1337.12
NW-Biotin	NWYLPWLGTNDWGGGK-Biotin	90.13	1044.26

Table S2. List of antibodies used in Western blotting

Antibodies	Species	Type	Dilution	Supplier	Catalogue number
Albumin	Rabbit	Polyclonal	1:5000	Proteintech	16475-1-AP
PHB1	Rabbit	Polyclonal	3:5000	GeneTex	GTX101105
PHB2	Rabbit	Polyclonal	3:5000	GeneTex	GTX102100
Anti-rabbit IgG (H+L), HRP	Donkey	Polyclonal	1:1000	Invitrogen	SA1-200
Anti-human IgG, HRP	Rabbit	Polyclonal	1:1000	Dako	P0214

Table S3. List of antibodies used in immunofluorescence studies

Antibodies	Species	Type	Dilution	Supplier	Catalogue number
Albumin	Rabbit	Polyclonal	1:500	Proteintech	16475-1-AP
Receptor tyrosine-protein kinase erbB-2	Rabbit	Polyclonal	1:500	Santa Cruz	sc284
Streptavidin, PE	-	-	1:300	BD Pharmingen™	554061
Anti-rabbit IgG, AF647	Donkey	Polyclonal	1:300	Abcam	Ab150075
Anti-human prohibitin	Mouse	Monoclonal	1:1000	Merk Millipore	MABE983
Anti-mouse IgG, FITC	Goat	Polyclonal	1:300	Sigma-Aldrich	F5387
